# Patched-2 functions to limit Patched-1 deficient skin cancer growth

**DOI:** 10.1007/s13402-018-0381-9

**Published:** 2018-06-04

**Authors:** Veronique L. Veenstra, Ilse Dingjan, Cynthia Waasdorp, Helene Damhofer, Allard C. van der Wal, Hanneke W. van Laarhoven, Jan Paul Medema, Maarten F. Bijlsma

**Affiliations:** 1 Laboratory for Experimental Oncology and Radiobiology, Center for Experimental and Molecular Medicine, Cancer Center Amsterdam and Academic Medical Center, Meibergdreef 9, 1105AZ, Amsterdam, The Netherlands; 2 Oncode Institute, Academic Medical Center, Amsterdam, The Netherlands; 3 Present Address: Tumor Immunology Lab, Radboud Institute for Molecular Life Sciences, Nijmegen, The Netherlands; 4 Present Address: Biotech Research & Innovation Centre, Copenhagen, Denmark; 5 Department of Pathology, Academic Medical Center, Amsterdam, The Netherlands; 6 Department of Medical Oncology, Cancer Center Amsterdam and Academic Medical Center, Amsterdam, The Netherlands

**Keywords:** Basal cell carcinoma, Hedgehog signaling, Patched, Smoothened

## Abstract

**Purpose:**

Basal cell carcinoma (BCC) is one of the most common skin cancers, and is typically driven by an aberrantly activated Hedgehog (Hh) pathway. The Hh pathway is regulated by interactions between the Patched-1 (Ptch1) and Smoothened (Smo) receptors. Smo is an activating receptor and is subject to inhibition by Ptch1. Following ligand binding to Ptch1, its inhibitory action is relieved and pathway activation occurs. This receptor interaction is pivotal to restraining uncontrolled cellular growth. Both receptors have been found to be frequently mutated in BCCs. Ptch2 is a Ptch1 paralog that exhibits overlapping functions in both normal development and tissue homeostasis. As yet, its contribution to cancer growth is poorly defined. Here we set out to assess how Ptch2 inhibits BCC growth.

**Methods:**

We used several in vitro readouts for transcriptional and chemotactic Hh signaling in BCC-derived ASZ001 cells, and a novel xenograft model to assess in vivo BCC tumor growth. Gene editing by TALEN was used to untangle the different Ptch2-dependent responses to its ligand sonic hedgehog (Shh).

**Results:**

We first defined the signaling competence of Ptch2 in Ptch1-deficient ASZ001 cells in vitro, and found that Ptch2 ligand binding drives their migration rather than eliciting a transcriptional response. We found that subsequent targeting of Ptch2 abrogated the chemotaxic effect. Next, we tested the contribution of Ptch2 to in vivo tumor growth using a xenograft model and found that reduced Ptch function results in increased tumor growth, but that selective pressure appatently acts against complete Ptch2 ablation.

**Conclusions:**

We conclude that like Ptch1, Ptch2 exerts a tumor-suppressive function in BCC cells, and that after targeting of both paralogs, ligand-independent activation of the Hh pathway contributes to tumor growth.

**Electronic supplementary material:**

The online version of this article (10.1007/s13402-018-0381-9) contains supplementary material, which is available to authorized users.

## Introduction

The Hedgehog (Hh) pathway is not only crucial to many inductive events in developing embryos and to the maintenance of tissue integrity in adult organisms, but also to the initiation and progression of tumors [1, 2]. Hh pathway regulation is primarily mediated by the transmembrane proteins Patched-1 (Ptch1) and Smoothened (Smo) [3, 4]. Ptch1 is the main receptor for the pathway-activating ligand sonic hedgehog (Shh) [5]. In the absence of this ligand, Ptch1 actively represses Smo to keep the pathway inactive [6]. In the presence of Shh the inhibitory action of Ptch1 is alleviated through its relocalization, and Smo is free to signal to downstream pathway components [7–9]. This intracellular signaling cascade can induce a variety of responses such as transcription factor activation and cytoskeleton remodeling to mediate chemotaxis [10, 11]. In cancer, two different mechanisms responsible for aberrant pathway activation can be discerned [12]. The first mechanism entails excessive production of Shh ligand by tumor cells, which subsequently acts in an autocrine or paracrine manner to provide tumor-promoting signals [13, 14]. The second mechanism entails genetic aberrations in Hh pathway components that cause Hh pathway activation [15–18]. These aberrations typically include inactivating mutations in Ptch1, crippling its inhibitory action on Smo, or activating mutations in Smo that render it insensitive to Ptch1 inhibition. Through these latter activating mutations pathway activity is induced cell autonomously, rendering the cells independent of Shh ligands produced by themselves or their surroundings. Given these considerations, Ptch1 is considered a bona fide tumor suppressor. One human cancer type that relies on mutations in Hh pathway components is basal cell carcinoma (BCC), the most prevalent skin cancer. Oncogenic mutations in Ptch1 and Smo are since long known to drive BCC, and mouse models have underscored the notion that the development of this malignancy relies heavily on these mutations [19, 20]. A recent study on the mutational profiles of 126 BCCs has revealed *PTCH1* mutations in 73% of the cases [21].

The current dogma on Hh pathway regulation holds that Ptch1 is the principal receptor for Shh, and that other receptors involved in Shh binding like Cdon, Boc and Gas1 function as coreceptors [22, 23]. A paralog of Ptch1 is Patched-2 (Ptch2) [24–26], and this paralog is thought to complement some Ptch1 functions [27–29]. It has been found, however, that Ptch2 does not act as an equally strong regulator of the pathway. For instance, *Ptch2*^*−/−*^ embryos have been found to be viable and to develop normally, and that in a genetically Ptch1-deficient system Ptch2 cannot fully compensate for loss of the other homolog [29–31]. However, Ptch2 deficiency does exacerbate the skin tumor phenotype in partially *Ptch1* deficient mice by deregulating epidermal lineage differentiation, and it has been found that the absence of both paralogs affects skin maintenance [32, 33]. Subsequent detailed analyses of Hh pathway target expression gradients in the epidermis revealed that full *Ptch* deficiency results in a uniformly high pathway activation [34]. Recent work in embryonic stem cells has shown that Ptch2 is required for ligand perception in the absence of Ptch1 [27]. Intriguingly, in a Ptch1-deficient mouse model of Hh pathway-driven BCC it was found that the tumors preferentially arise from locations close to Shh sources [19]. These latter observations imply that in the absence of Ptch1 at least some responsiveness to Shh remains and, therefore, that Shh is a likely candidate to mediate Ptch2 activity.

*PTCH2* mutations are relatively rare events. A large-scale genetic analysis revealed that only 14 out of 126 BCC cases carried mutations in both *PTCH1* and *PTCH2* and that only 4 cases exclusively carried *PTCH2* mutations [21]. These observations suggest that in the absence of functional PTCH1, there is little selective pressure on PTCH2. Here, we asked whether absence of repressive PTCH1 action enhances the role of PTCH2 in Shh ligand perception and subsequent pathway activation, rendering cells highly sensitive to Shh ligand, or whether the contribution of PTCH2 to tumor growth is solely dependent on its tumor suppressor function via the suppression of Smo activity. Another question to be answered is whether there is selective pressure against ablation of both *PTCH* paralogs, which might explain the low incidence of *PTCH2* mutations observed in patient samples. We used in vitro and in vivo systems, in conjunction with gene editing, to untangle the different responses of BCC cells to Shh ligand and show that deficiency for both *PTCH* paralogs accelerates tumor growth.

## Materials and methods

### Cell culture

PANC-1 cells (ATCC, Manassas, VA) and mouse embryonic fibroblasts (*Ptch1*^*−/−*^ and *Ptch1*^*+/+*^ MEFs from Dr. Scott, Stanford University [35]) were cultured in high-glucose DMEM containing 8% fetal bovine serum (FBS), L-glutamine, penicillin and streptomycin (all from Lonza, Basel, Switzerland) according to routine cell culture procedures. ASZ001 cells [36, 37] were cultured in 154CF keratinocyte medium (Life Technologies) supplemented with 50 μM CaCl, penicillin and streptomycin, and 2% chelex-treated FCS. Cells were screened for mycoplasma monthly by PCR.

### Quantitative RT-PCR

Cells were lysed in Trizol (Invitrogen) after which RNA was isolated according to the manufacturer’s protocol. cDNA was synthesized using Superscript III (Invitrogen) and random primers (Invitrogen). Quantitative real-time RT-PCR (qRT-PCR) was performed using SYBR green (Roche, Basel, Switzerland) on a Lightcycler LC480 II (Roche). Relative gene expression levels were calculated using the comparative threshold cycle (Ct) method and values were normalized to the reference gene *Gapdh*. The primer sequences used were: *Gapdh* 5’ CTCATGACCACAGTCCATGC and 3’ CACATTGGGGGTAGGAACAC; *Gli1* 5’ ATAGGGTCTCGGGGTCTCA and 3’ CGGCTGACTGTGTAAGCAGA; *Ptch1* 5’ GCTACGACTATGTCTCTCACATCAACT and 3’ GGCGACACTTTGATGAACCA; *Ptch1* (exon 2) 5’ CTGTGGCTGAGAGCGAAGTT and 3’ AGCTCCTCCACGTTGGTCT; *Ptch2* 5’ GCGTACACCTCCCAGATGTT and 3’ GGAACCCCTGATTTGTAGCA.

### FACS analysis

Cells were harvested using a trypsin-EDTA solution (Lonza) and washed in FACS buffer (PBS containing 1% FBS). Hybridoma supernatants containing either anti-Shh antibody 5E1 [38] or anti-Myc antibody 9E10 (isotype) were diluted 1:5 in FACS buffer and incubated for 30 min at 4 °C. A secondary APC labeled anti-mouse (BD, 550826) antibody was used at a dilution of 1:500. After washing, the cells were resuspended in FACS buffer containing 1 μg/ml propidium iodide (PI) (Sigma) and subjected to flow cytometry using a FACSCanto II machine (BD, Franklin Lakes, NJ, USA). The data obtained were analyzed using FlowJo 7 software (Tree Star, Ashland, OR, USA).

### Immunofluorescence

ASZ001 cells were grown on glass coverslips, starved for 2 d, and fixed using 4% formaldehyde. Following blocking and permeabilization in 5% goat serum/phosphate-buffered saline with 0.1% Triton X100 (PBS-T), a primary antibody directed against acetylated α-tubulin (Sigma) was added at 1:2000 and incubated for 1 h at room temperature or overnight at 4 °C. Next, an Alexa 488 conjugated anti-mouse secondary antibody (Invitrogen) was added at 1:2000 and incubated for 1 h at room temperature. Finally, the coverslips were mounted using ProLong Gold (Invitrogen) and images were captured using a Zeiss AxioVert microscope. For Smo ciliary localization, cells were transfected with Myc-tagged Smo using PEI 48 h prior to starvation and ShhN stimulation (1:4 diluted supernatant from 293 T cells).

### GBS-GFP reporter construct and cell line establishment

A concatemerized 8 × 3’GLI binding site sequence (GBS) was isolated from the pδ51 GBS-luciferase reporter construct [39] by PCR and cloned into a lentiviral pRRL TOP-d2GFP reporter vector [40] as reported before [41]. MEFs and ASZ001 cells were transduced with the GBS-GFP reporter construct, starved in 0.5% FCS containing medium and stimulated for 4 d with ShhN conditioned supernatant from 293 T cells or 200 nM Smo agonist (SAG, EMD Millipore, Billerica, MA, USA). The resulting cells were sorted on a BD FACSAria for GFP expression, after which GFP positive cells were grown under 8% FCS (MEFs) or 2% FCS (ASZ001) conditions, resulting in the expected loss of GFP activity. For subsequent analyses, cells were seeded in 24-well plates and treated as indicated in the figure panels. Following treatment, cells were harvested and the percentages of GFP^+^ cells were determined by flow cytometry on a FACSCanto II machine.

### Cell viability assays

Cells were seeded at 2000 cells/well in 0.5% or 2% FCS and after 3 h treated with the indicated compounds (see also [42]). After 4 d, MTT was added and dye reduction was assessed after a 4 h incubation period. Background (10 mM H_2_O_2_ treated cells) levels were subtracted and the values obtained from the control treated cells were set to 1, after which the experimental data were normalized to the controls.

### Transwell migration assays

Migration assays were performed as previously reported [10]. Briefly, cells were labeled with 10 μM CellTracker Green (Invitrogen) according to the manufacturer’s protocol. After labeling, the cells were detached using 5 mM EDTA, resuspended in serum free medium and transferred to FluoroBlok Transwell inserts (BD Falcon) at a density of approximately 5 × 10^4^ cells per insert. Chemoattractant was added to the bottom compartments of the Transwell plates and GFP-spectrum fluorescence in the bottom compartments was measured using a Synergy HT plate reader (BioTek, Winooski, VT, USA) every 2 min during approximately 3 h. Background fluorescence was measured in time from a well containing only medium and these values were subtracted from all other measurements. The ‘no attractant’ control was used to measure baseline cell movements for every experimental condition. These values were subtracted from those obtained in the presence of chemoattractants in the bottom compartments of the Transwell inserts. The resulting data yielded specific migration values towards a given attractant.

### Gene editing and transfections

The pCTIGTALEN expression vector was used as described before [27]. A pair of TALEN constructs was modified so that one construct co-expressed GFP and the other tdTomato, allowing for selection for both 5′ and 3′ targeting constructs by FACS sorting. The constructs were designed using Golden Gate cloning into pCTIG employing the following variable domain architectures: 5’ NN NN HD NG NG HD NN NI NN HD NG NG NI HD NG NG HD; 3’ NG HD NG NN NN NI NG HD HD NG NN HD NI HD HD HD HD. See also Supplementary Fig. S4a.

ASZ001 cells grown in 3 × 12-well plates were transfected with paired TALEN constructs using PEI. 3 days after transfection GFP^+^/tdTomato^+^ cells cells were selected by flow sorting, seeded in bulk and allowed to recover for 7 d in T25 flasks. To obtain monoclonal cultures, cells were seeded at single cell/well densities in 2 × 96-well plates. In 35 of the 192 wells cells grew out. These cells were genotyped through the sequencing of PCR products spanning the TALEN binding sites (see also Supplementary Fig. S5). Screening was performed on genomic DNA by PCR using primers flanking the TALEN binding sites: 5’ AAGGCACAGGGAAAGAGAGTT; 3’ ACTTGCCTAGCTTGCACAATG and subsequent digestion of the PCR products with AccI. Genomic DNA from monoclonal lines that exhibited loss of the restriction site were TOPO cloned (Thermo Fisher) and Sanger sequenced.

Overxpression of Hedgehog pathway components was accomplished using wild-type *Smo* (*SmoWT*) and ciliary localization domain mutated *Smo* (*SmoCLD*) constructs in *pCS107* obtained from Dr. Jeremy Reiter [7, 43] and a wild-type *Ptch1* construct in *pcDNA3.1* obtained from Dr. Matthew Scott (see also ref. [44]). *mPtch1Δ*^*loop2*^ is a Ptch1 form that lacks the second extracellular loop required for ligand binding and only exerts Smo-repressive functions [45].

### Xenografting of ASZ001 cells

NOD.Cg-*Prkdc*^*scid*^*Il2rg*^*tm1Wjl*^/SzJ (NSG) mice were bred in-house. The animals were grafted with 1 × 10^5^ or 5 × 10^5^ ASZ001 cells in Matrigel [46]. All experiments were performed according to procedures approved by the animal experiment ethical committee (LEX237). Tumor growth was monitored weekly and the experiments were ended when ulceration was observed. Of note, ulceration was typically observed before the tumors reached a humane endpoint volume (1000 mm^3^). Finally, the tumors were harvested and processed for paraffin embedding and subsequent histopathological examination, as well as for RNA extraction.

## Results

### Ptch1-deficient basal cell carcinoma cells perceive hedgehog signalling

**Fig. 1:**
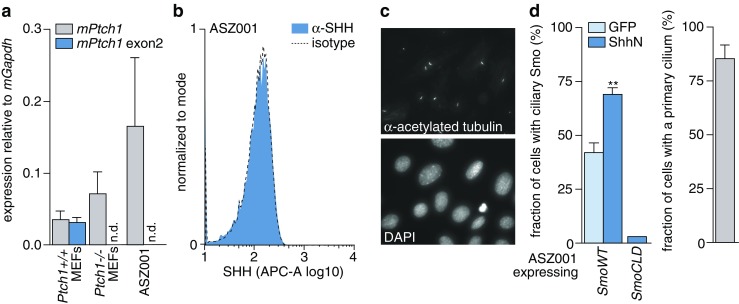
Ptch1-deficient BCC cells perceive Shh ligand. **a** RNA was isolated from the cells indicated on the X-axis after which qRT-PCR-based expression analysis was performed relative to *mGapdh* using a *Ptch1* exon upstream of the targeted exon (grey bars) and the targeted exon 2 (dark blue bars). Bars indicate means ± SEM (*n* = 4), n.d. indicates not detected (no signal). **b** ASZ001 cell surface levels of Hedgehog ligands were determined by FACS using a 5E1 anti-Shh hybridoma antibody or an isotype control. **c** ASZ001 cells were grown to confluence on coverslips after which primary cilia were visualized by acetylated α-tubulin staining. Nuclei were counter-stained with DAPI. **d** ASZ001 cells were grown on coverslips, transfected with Myc-tagged forms of *Smo*, starved and treated with ShhN (or control; GFP) supernatant diluted 1:4 from 293 T cells for 1 h. Next, the cells were fixed and stained for Myc and acetylated α-tubulin, after which the percentage of transfected (Myc-positive) cells with ciliary Smo was quantified. *SmoCLD*; ciliary localization domain mutated form of Smo [7, 43]. For assessment of the fraction of ciliated cells, quantifications from both transfections were pooled and depicted in the separate grey bar graph. Bars indicate means ± SEM (*n* > 50 cells quantified from 2 separate experiments). **p = 0.0015 by Mann-Whitney U test

As a model for Ptch1-deficiency driven skin cancer, we used the ASZ001 cell line. This cell line has been established from an irradiation-induced tumor in a *Ptch1*^*+/-*^ mouse, and has subsequently undergone *Ptch1* loss of heterozygosity ( [36] and Fig. 1a). The current working model for Hedgehog (Hh) pathway regulation dictates that loss of Ptch1 suffices for full Hh pathway activation and that no additional ligand is required for this. Indeed, the Hh pathway was found to be activated in these cells as evident from abundant transcription of the remaining exon on the targeted *Ptch1* allele, which itself is a target of the activated pathway (Fig. 1a). We also found that the cells did not produce ligand that could cell autonomously activate the pathway (Fig. 1b; SHH expressing pancreatic cancer PANC-1 and *Shh* transfected ASZ001 cell controls are shown in Supplementary Fig. S1). One prerequisite for Hh ligand responsiveness, i.e., the presence of a primary cilium, was confirmed in nearly all ASZ001 cells analyzed (Fig. 1c, right panel) [7]. The primary cilium is an antenna-like protrusion from the cell membrane that is shaped by the microtubule cytoskeleton. A dynamic localization of Hh pathway components in and out of this organelle is required for pathway regulation, and the primary cilium is required for appropriate ligand perception. Thus, at least part of the immediate signaling machinery for Shh is intact in these cells. The ciliary localization of exogenously overexpressed Smo in response to the addition of Shh was subsequently assessed by microscopy. Despite a high baseline percentage of cells with Smo localized in the cilium, as expected from a Ptch1-deficient system and overexpression of Smo, we found that the addition of ligand resulted in a moderate increase in this number (Fig. 1d). As a control, a form of Smo that cannot localize to the cilium (*SmoCLD*), was used [7]. Taken together, our results imply that in the absence of Ptch1, the Shh ligand is still perceived by the cells.

### ASZ001 cells respond to hedgehog by chemotaxis

**Fig. 2:**
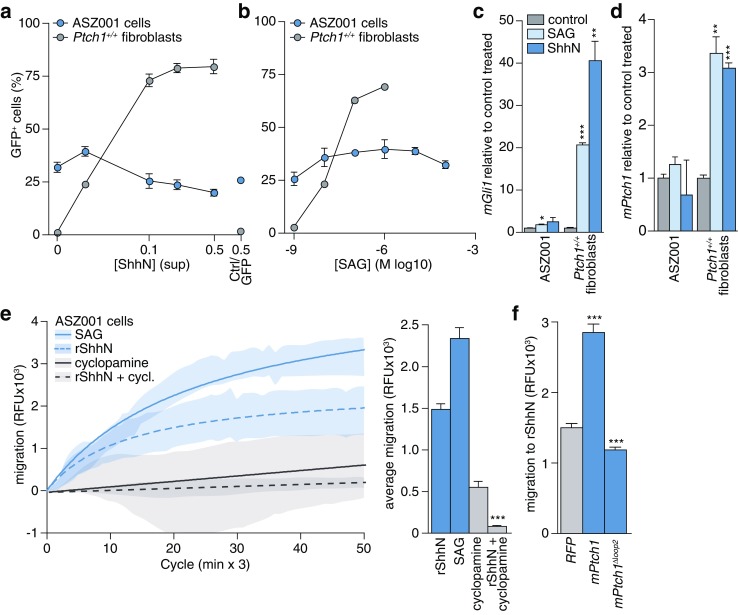
ASZ001 cells mediate a chemotactic response to Shh. **a** GBS-GFP transduced ASZ001 and Ptch1-proficient fibroblasts were starved and treated with the indicated dilutions of ShhN supernatants produced by 293 T cells. For the highest concentration, GFP transfected 293 T supernatant was included as a control (Ctrl/GFP 0.5). After 3 d, the GFP^+^ percentage was assessed by FACS (*n* = 4 for the ASZ001 cells; *n* = 2 for the MEFs). **b** As for panel a, using SAG. At the two highest tested doses, SAG was toxic to the fibroblasts (*n* = 3 for the ASZ001 cells; *n* = 4 for the MEFs). **c-d** Cells were treated as for panels a-b using 200 nM SAG or 1:4 diluted ShhN supernatant. After treatment, RNA was isolated and qRT-PCR was performed for *mGli1* and *mPtch1* (*n* = 5). *p < 0.05; **p < 0.01; ***p < 0.001; determined by t-test, (**e**) ASZ001 cells were seeded in a modified Boyden chamber after which net migration to 5 nM recombinant ShhN or 200 nM SAG was assessed in the absence or presence of 5 μM cyclopamine in both the upper and the bottom compartments of the Transwells. Shaded curves represent the SEM. The curves were plotted using a hyperbola function. Measurements from at least 3 replicates are shown. For details see Materials and methods section. The data are plotted as average RFU in bar graphs. Statistical test compares rShhN + cyclopamine versus rShhN. The difference in migration between no-attractant control and ShhN/SAG is statistically significant (*p* < 0.001). **f** ASZ001 cells were transfected with indicated constructs, and after ~24 h the migration response to 5 nM recombinant ShhN was assessed. Bars indicate means ± SEM from approximately 70 measurements from 2 separate experiments. Differences to the vector control were tested for the *mPtch1* transfected condition, and for the *mPtch1* condition against the *mPtch1*^***Δ****loop2*^ condition

Typically, relocalization of Smo to the primary cilium results in activation of the downstream pathway leading to transcriptional responses. However, when Gli transcription factor activity was measured in these cells using a stably integrated GLI-binding site GFP reporter construct, only minimal responses to exogenous pathway activators were observed, although the baseline pathway activity was high (Fig. 2a,b). In comparison, MEFs proficient for Ptch1 showed robust responses to the pathway activators. These results were confirmed by qRT-PCR analysis of target genes, through which minor responses were observed in ASZ001 cells compared to MEFs (Fig. 2c,d, for baseline pathway activity see Fig. 1a). Under high serum proliferation conditions, a strong reduction in basal reporter activity in the ASZ001 cells was observed (4% GFP^+^ cells, data not shown), indicating that the Hh pathway in these cells is amenable to at least some degree of regulation, most likely through cell cycle progression and subsequent primary cilium loss. Switching these cells back to low-serum conditions resulted in a regain of GFP reporter expression to the levels shown in Fig. 2a,b. In order to test whether the mitogenic Hh response can be driven by exogenously added ligand in ASZ001 cells, we treated them with ShhN or control (Ctrl/GFP) supernatants. By doing so, we observed only marginal proliferative responses to Shh compared to the control (Supplementary Fig. S2a). In addition, we found that inhibitors of Hh pathway components and related signaling molecules were relatively ineffective in restraining ASZ001 cell proliferation, as evident from their overall high IC50 values (Supplementary Fig. S2b,c) [42, 44, 47].

As shown previously in fibroblasts and neural developmental models, Shh ligand is also able to induce chemotaxis, irrespective the presence of Hh pathway transcription factors. This response appears to be relatively resilient to the perturbation of pathway components [43]. To assess whether the perception of Shh by ASZ001 cells may function to activate this chemotactic response, we assessed the migratory capacity of these cells. Pathway activators were used in a modified Boyden chamber assay to quantitatively measure chemotaxis. A robust migratory response was observed to both recombinant ShhN (5 nM) and SAG (200 nM; Fig. 2e). This response could be blocked with cyclopamine (5 μM), confirming that this form of chemotaxis requires Smo. The subsequent use of a Smo agonist (SAG) further underscored the Smo dependency of this migratory response. After exogenous Ptch1 overexpression, we observed an increased relative responsiveness of the cells to ligand as evident from an increased net migratory response to Shh (Fig. 2f). A form of Ptch1 that is unable to bind Shh (Ptch1^Δloop2^) was found to be inhibitory to the Shh stimulated migration [45]. This suggests that the migratory response, although not strictly dependent on Ptch1, can be modulated by the inhibitory activity of Ptch1. Together, these data indicate that despite the absence of Ptch1, ASZ001 cells have retained Shh chemotactic responsiveness. This notion suggests that other receptors may mediate the chemotactic response to Shh. A likely candidate to mediate this response is its paralog Ptch2.

### Ptch2 mediates a chemotactic response to hedgehog ligand

Due to the catalytic inhibitory activity of Ptch on Smo, a low number of Ptch molecules suffices to suppress Hh pathway activity [6] and, therefore, highly effective targeting is required to study the consequences of Ptch loss. We found that lentiviral shRNA-mediated Ptch2 silencing using five different sequences and puromycin selection did not result in effective targeting (Supplementary Fig. S3). Therefore, we turned to transcription activator-like effector nucleases (TALENs) to edit the *Ptch2* locus in ASZ001 cells (strategy shown in Supplementary Fig. S4). Following transfection, cells were FACS-sorted in bulk to ensure the survival of cells expressing both TALENs (Supplementary Fig. S5a). After recovery, the cells were seeded at single cell density in 2 × 96 well plates after which 35 of the clones grew out (18%). These single cell clones were analyzed for editing events and, by doing so, we found that in 5 of the clones the digest pattern was indicative of an editing event (Supplementary Fig. S5b; lines 1, 4, 14, 16, 17). These lines were TOPO cloned after which 20 clones per line were Sanger sequenced to verify the efficiency of editing (Supplementary Fig. S5c). Surprisingly, none of these lines was completely devoid of wild type *Ptch2* sequences. In cell line 4, one third of the sequences were mutant, whereas in cell lines 1, 14, 16 and 17 two-thirds of sequences were mutant. In addition, we found that the patterns of gene editing in cell lines 1, 14, 16 and 17 were nearly identical, suggesting that amongst the initial 35 cell lines available for restriction digest analysis, only two parental clones showed significant *Ptch2* editing, i.e., clone 4 (denoted *Ptch2*^*MED*^) and an additional parental clone which gave rise to clones 1, 14, 16 and 17 (denoted *Ptch2*^*LOW*^), of which we continued with clone 1. The failure to establish cell lines with complete *Ptch2* gene editing from otherwise successfully transfected cells implies that selective pressure exists against full Ptch-deficiency. This notion is in agreement with the lentiviral shRNA *Ptch2* silencing results (see Supplementary Fig. S3).

**Fig. 3:**
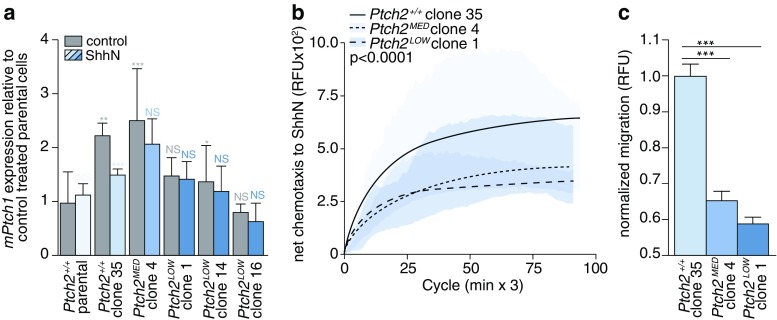
Ptch2 is required for Shh chemotaxis. **a** ASZ001 *Ptch2*^*+/+*^ parental cells and TALEN genome edited ASZ001 cells were stimulated with ShhN as depicted in Fig. 2c-d. Target gene (*mPtch1*) transcript analysis was performed. Genotypes and clones are indicated on the X-axis and by the blue-shaded (ShhN) bars. Blue asterisks denote statistical comparisons of ShhN-treated cells and control treated cells of the same genotype. Grey asterisks indicate significance compared to ASZ001 *Ptch2*^*+/+*^ parental cells. *p < 0.05; **p < 0.01;***p < 0.001; determined by t-test, (**b**) *Ptch2*^*+/+*^, *Ptch2*^*MED*^*,* and *Ptch2*^*LOW*^ cells were seeded in a modified Boyden chamber after which specific migration (net chemotaxis) to 5 nM recombinant ShhN was assessed as in Fig. 2e. Measurements from 4 replicates in 2 experiments are shown. The indicated *p*-values were determined by one-way ANOVA. **c** Schematic representation of Fig. 3b. Significances were determined using Mann-Whitney U test

Cells targeted for Ptch2 were found to be transcriptionally unresponsive to ShhN ligand as determined by qRT-PCR (Fig. 3a). Surprisingly, we also found that *Ptch2* gene editing resulted in an increase in basal transcriptional pathway activity in vitro when comparing wild-type Ptch2^+/+^ parental cells with wild-type Ptch2^+/+^ clone 35, arguing for the use of gene edited but wild-type cells as controls. If Ptch2 mediates chemotaxis in response to Hh ligand as was concluded from the data presented in Fig. 2, its targeting should inhibit the chemotactic response. Indeed, we observed a strong reduction in chemotaxis to ShhN in the Ptch2^LOW^ and Ptch2^MED^ cells, as compared to the Ptch2^+/+^ clone 35 cells (Fig. 3b-c). Baseline chemokinesis (i.e., the movement of cells in the absence of attractant; these values are typically subtracted from migration to Shh to yield net chemotaxis as shown in Fig. 3b) did not differ between the genotypes (Supplementary Fig. S6a). The residual chemotactic responsiveness of *Ptch2*-edited ASZ001 cells was sensitive to cyclopamine, showing that the regulation of Shh chemotaxis is dependent on Smo (Supplementary Fig. S6b).

Also, receptors other than Ptch1 and Ptch2 have been implicated in the perception of Shh for chemotactic responses [23, 48, 49]. In order to test whether putative Hh receptors in addition to Ptch2 may mediate Shh chemotaxis in ASZ001 cells, we targeted the Cdon and Boc receptors by lentiviral shRNA delivery and found that, despite a modest knockdown efficiency, migration was hampered (Supplementary Fig. S6c,d). This implies that in addition to Ptch2, other candidate receptors for Shh may elicit chemotactic responses. Nevertheless, given that Ptch2 is incapable of mediating a robust transcriptional response to ShhN (Fig. 2a-d and 3a), we conclude that the ligand-dependent contributions of Ptch2 in Ptch1-deficient cells in vitro are confined to the chemotactic response.

### Ptch2 deficiency accelerates tumor growth

**Fig. 4:**
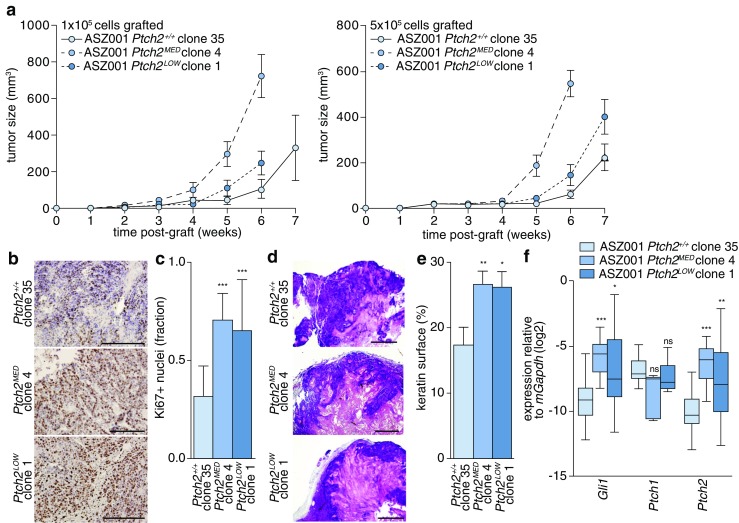
Patched deficiency accelerates tumor growth. **a** 1 × 10^5^ and 5 × 10^5^ ASZ001 cells of the indicated genotypes were subcutaneously grafted in immune deficient mice in PBS/Matrigel. Subsequent tumor growth was monitored and tumors were harvested after signs of ulceration. In each group 4 mice were grafted with 2 tumors, yielding sample sizes of 8. **b** After harvesting, the tumors were processed for Ki67 immunohistochemistry. Scale bars: 200 μm. **c** Automated quantification of positive nuclei per optical field (*n* ≥ 7). The indicated *p*-values were determined using Mann-Whitney U test. **d** Histology of tumors grown from the indicated genotypes revealed by hematoxylin and eosin staining. Scale bar: 1 mm. **e** Automated quantification of pink eosin-stained fields shown in d. **f** After RNA extraction from the harvested tumors transcript analyses for the indicated genes were performed (*n* = 16). Significances were determined using Mann-Whitney U test (see Fig. 3)

Having established that (1) in the absence of Ptch1 Hh ligand is perceived by Ptch2 only to mediate chemotaxis and (2) that additional targeting of Ptch2 impedes the ability of the cells to perceive Hh ligand, we proceeded to test the consequences of these signaling outputs for in vivo tumor growth. To this end, ASZ001 cells were injected subcutaneously in immunodeficient mice after which tumor growth was monitored. We found that tumors grown from cells targeted for both Ptch paralogs (i.e., *Ptch2*^*LOW*^ and *Ptch2*^*MED*^ ASZ001 cells) expanded faster than those grown from fully Ptch2-proficient cells (i.e., *ASZ001 Ptch2*^*+/+*^ clone 35 cells). The former mice were sacrificed sooner based on ulceration (Fig. 4a). Subsequent immunohistochemical analysis for the proliferation marker Ki67 confirmed a higher proliferative index in the *Ptch2* gene-edited tumors (Fig. 4b, quantification in Fig. 4c).

Assessment of tumor histology by a pathologist confirmed a cutaneous source of the tumor cells, but also revealed a squamous rather than basal histology for both genotypes, possibly owing to extensive in vitro culturing of the cells prior to grafting. The *Ptch2*^*LOW*^ and *Ptch2*^MED^ derived tumors showed more keratin depositions (eosin rich pink areas in Fig. 4d; quantification in Fig. 4e). To determine whether the in vivo accelerated proliferation of these *Ptch2* gene-edited cells could be explained by additional activation of the Hh pathway over the already high level caused by the Ptch1-deficiency, the expression of Hh pathway target genes was measured by qRT-PCR. Indeed, these were found to be elevated in the *Ptch2*^*LOW*^ and *Ptch2*^MED^ ASZ001 tumors (Fig. 4f) and are likely responsible for the observed increase in tumor growth rates. We hypothesize that the discrepancy in Hh pathway activation in vitro and in vivo following Ptch2 targeting results from environmental signals and/or mechanical properties that are only present in vivo, which feed into the signaling cascade downstream of Smo. From the increased baseline pathway activity and associated tumor growth following Ptch2 targeting in vivo, we conclude that Ptch2 exerts ligand-independent pathway inhibitory functions that make it a tumor suppressor, but one that is also required and essential for cancer cell viability.

## Discussion

The current model of Hedgehog (Hh) pathway regulation holds that Ptch1 acts as the main receptor for Shh, and that it serves as the master switch for downstream pathway regulation [3, 6]. Recent work has shown, however, that the concept of Ptch1 as key Shh receptor needs revision. In the absence of Ptch1, Ptch2 has been found to be required for the perception of ligand in developmental models [27]. Also, Ptch1 and Ptch2 have been shown to exhibit overlapping functions in Hh pathway-dependent skin development and maintenance [32, 34]. The exact contribution of Ptch2 to an established cancer type such as basal cell carcinoma (BCC) has, however, remained unclear, despite clinical data that suggest a tumor suppressor function [50]. Whether such a putative tumor suppressor function of Ptch2 may be uncoupled from its ligand binding function is currently unknown and is possibility complicated by the fact that these functions are connected [45]. Here, we have untangled these functions by first delineating the signaling capabilities of Ptch2 in BCC cells in vitro and subsequently testing the consequences of Ptch2 perturbation for in vivo BCC tumor growth. Using migration assays we found that in the parental *Ptch1*^*−/−*^ ASZ001 cells the only response to Hh ligand was chemotactic. After *Ptch2* targeting by TALEN we found that this chemotactic response was severely hampered. Therefore, we conclude that the increased tumor growth observed following targeting of Ptch2 is likely unrelated to ligand perception and that the tumor suppressive action of Ptch2 very likely depends on its baseline inhibitory activity towards Smo, which for an as yet unknown reason only becomes apparent in vivo*.*

As mentioned above, *Ptch2* mutations are uncommon in BCC [21]. This is at apparent odds with its similarities and overlapping signaling roles with Ptch1. However, as we find that Ptch2 has a very limited ligand-perceiving role in BCC, we assume that its main function as a tumor suppressor is overshadowed by the loss of Ptch1, which is a stronger inhibitor of the Hh pathway [31, 33]. This notion is also supported by the high incidence of mutations in for instance *TP53*. TP53 is a much stronger tumor suppressor than Ptch2, and it is more easily perturbed given that only one allele requires a mutation to yield an oncogenic event. Mutations in *TP53* are, therefore, more advantageous and more regularly achieved than *PTCH2* LOH which, in turn, could explain the low mutation rate of *PTCH2*. Another explanation for the paucity of *PTCH2* mutations in human BCC follows directly from our observation that full *Ptch2* gene ablation could not be achieved, despite selection. It thus appears that a low level of Ptch2 activity is required for cancer cell viability, at least in vitro. Modest perturbations of Ptch2 are apparently tolerated, resulting in sufficient Hh pathway upregulation in vivo to boost tumor growth, as evident from the enhanced tumor growth found for the *Ptch2*^*MED*^ cells. How these two counteracting activities are balanced remains to be determined, but it is possible that they are driven by distinct signaling functions of Ptch2 (Smo antagonist, chemotaxis receptor, dependence receptor), or by a cancer-specific context. In addition, it is possible that rare tumors that are currently not recognized to be Hh-driven may rely on mutations in Ptch2 if that serves as the dominant Hh receptor in the tissue of origin. For instance, the testis-specific expression of Desert Hedgehog (Dhh) and Ptch2 and their role in glioblastoma-endothelium crosstalk hints to this possibility [24, 51, 52]. We found that some chemotactic responsiveness was retained by the Ptch-edited ASZ001 cells. We hypothesize that this responsiveness may result from candidate coreceptors for Shh other than Ptch2, such as Cdon, Boc or Gas1, or possibly Smo itself [53]. Indeed, we found that the Cdon and Boc receptors may also contribute to chemotactic Shh signaling. Given the fact that the only experimental variable in the xenografting experiments was the level of Ptch2 we are, however, reluctant to draw firm conclusions on its signaling contributions in vivo.

One caveat of our study is that it is yet unclear to what extent our findings in murine cells can be translated to the human situation. The ASZ001 cell line represents a powerful exerimental tool given that these cells are unambiguously Hh-dependent, which is important for our experiments, and that many murine-specific genetic tools to study Hh signaling are available. Nevertheless, validation in a human-derived model is warranted. In addition, it remains to be established whether the identified tumor suppressive action of Ptch2 is also relevant for non- or pre-malignant cultured keratinocytes.

Previously, we have shown that ciliary relocalization of Smo in response to Shh is not required for Shh chemotaxis, and suggested that chemotactic and transcriptional responses to Shh may represent separate phenomena resulting from distinct intracellular mechanisms [43]. Our current data indicate that these responses are indeed not mutually exclusive, and that Smo localization to the cilium occuring in the absence of robust downstream transcriptional signaling (i.e., in *Ptch1*^*−/−*^*;Ptch2*^*+/+*^ cells) may initiate a chemotactic response. In fact, the convergence of both pathways at several points within the signaling cascade (Smo, the primary cilium) seems to suggest that they are both part of a relatively conserved pathway. This holds promise for the application of currently available drugs against BCC such as erivedge/vismodegib [54]. Given the fact that vismodegib acts to inhibit Smo, rather than downstream signaling components or transcription factors, it may be effective against both the chemotactic and the transcriptional ligand responses, as well as against baseline pathway activity. This drug may, therefore, turn out to be effective against BCC irrespective the mutational status of other Hh ligand receptors.

## Electronic supplementary material

ESM 1 (DOCX 1.11 mb)
