# Supplementary material for: Patched-2 functions to limit Patched-1 deficient skin cancer growth
Source: Cell Oncol (Dordr). 2018 Jun 4;41(4):427–37. doi: 10.1007/s13402-018-0381-9 (PMC6105176; doi:10.1007/s13402-018-0381-9)
Supplement: Supplementary file 1 — (DOCX 1.11 mb) [file 13402_2018_381_MOESM1_ESM.docx]

### Supplemental material for

# Patched-2 functions to limit Patched-1-deficient skin cancer growth

Veronique L. Veenstra^1,2^, Ilse Dingjan^1,3^, Cynthia Waasdorp^1^, Helene Damhofer^1,4^, Allard C. van der Wal^5^, Hanneke W van Laarhoven ^6^, Jan Paul Medema^1,2^, and Maarten F. Bijlsma^1,2^

^1^Laboratory for Experimental Oncology and Radiobiology, Center for Experimental and Molecular Medicine, Cancer Center Amsterdam and Academic Medical Center, Amsterdam, The Netherlands

^2^Oncode Institute, Academic Medical Center, Amsterdam, The Netherlands

^3^Current address: Tumor Immunology Lab, Radboud Institute for Molecular Life Sciences, Nijmegen, The Netherlands

^4^Current address: Biotech Research & Innovation Centre, Copenhagen, Denmark

^5^Department of Pathology, Academic Medical Center, Amsterdam, The Netherlands

^6^Department of Medical Oncology, Cancer Center Amsterdam and Academic Medical Center, Amsterdam, The Netherlands

**Corresponding Author**: Maarten F. Bijlsma, Laboratory for Experimental Oncology and Radiobiology, Center for Experimental and Molecular Medicine, Cancer Center Amsterdam and Academic Medical Center, Meibergdreef 9, 1105AZ Amsterdam, The Netherlands. E-mail: m.f.bijlsma@amc.uva.nl

###

### Supplementary Fig. 1 FACS based detection of Hedgehog ligands. (a) Human PANC-1 pancreatic cancer cells that endogenously express high levels of SHH protein were used to validate the FACS based measurement of Hedgehog ligands. Cells silenced for SHH (*shSHH*) were used as a control for specificity, while 9E10 anti-Myc hybridoma supernatant was used as an isotype control. (b) ASZ001 cells were transfected with wildtype *Shh* (*ShhWT*; full-length), or the C-terminal domain (*ShhC*) against which the 5E1 antibody is not reactive. These results show that ASZ001 are capable of expressing forms of Hedgehog ligand that can be detected by FACS. Of note, the percentage of Shh-positive cells exceeds that of the transfection efficiency (cf. Supplementary Figure 5a), presumably caused by transfer and membrane retention of the hydrophobic Shh protein.

###
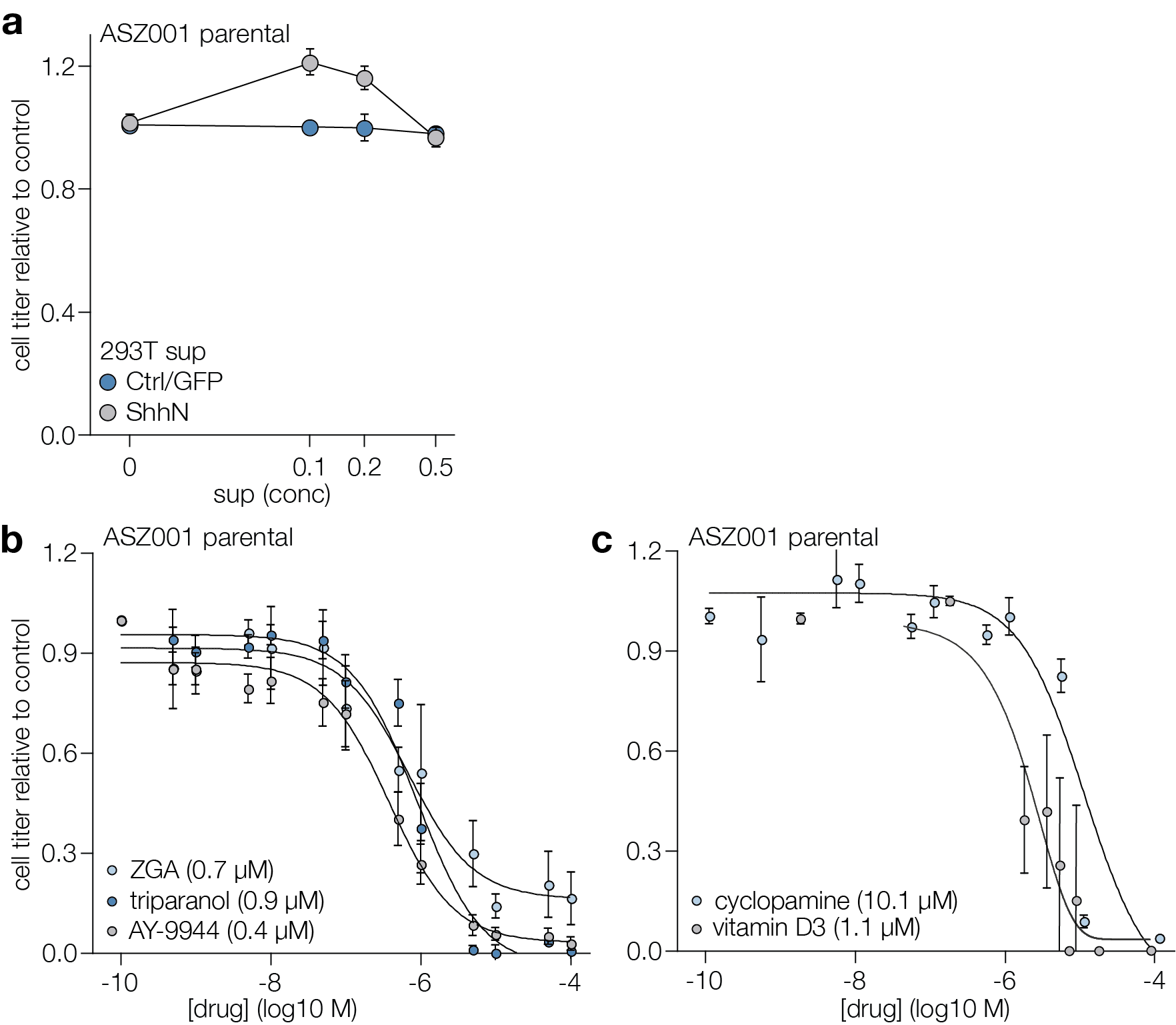


### Supplementary Fig. 2 ASZ001 cell viability does not rely on extrinsic Hedgehog pathway activators. (a) 293T cells were transfected with a soluble form of *Shh* (*ShhN*) or GFP control and after 72 h, (154CF medium) supernatant was harvested and used to treat ASZ001 cells. ASZ001 cells were plated at 2000 cells per well in 0.5% FCS and 4 h after plating, cells were treated with 293T supernatant diluted 1:4. After 72 h viability was determined by MTT assay. (b) ASZ001 cells were plated and treated as for panel a, with indicated sterol synthesis inhibitors. Numbers in parentheses indicate IC50. (c) As for panel b, using indicated inhibitors of Smo.

**Supplementary** **Fig. 3** Lentiviral shRNA silencing of Ptch2 in ASZ001 cells. Lentivirus was produced using third-generation helper plasmids and pLKO.1 vectors from the TRC Mission shRNA library. Puromycin selection was applied until control cells had all died, and qRT-PCR for *mPtch2* and *mGapdh* was performed on the selected cells.

**
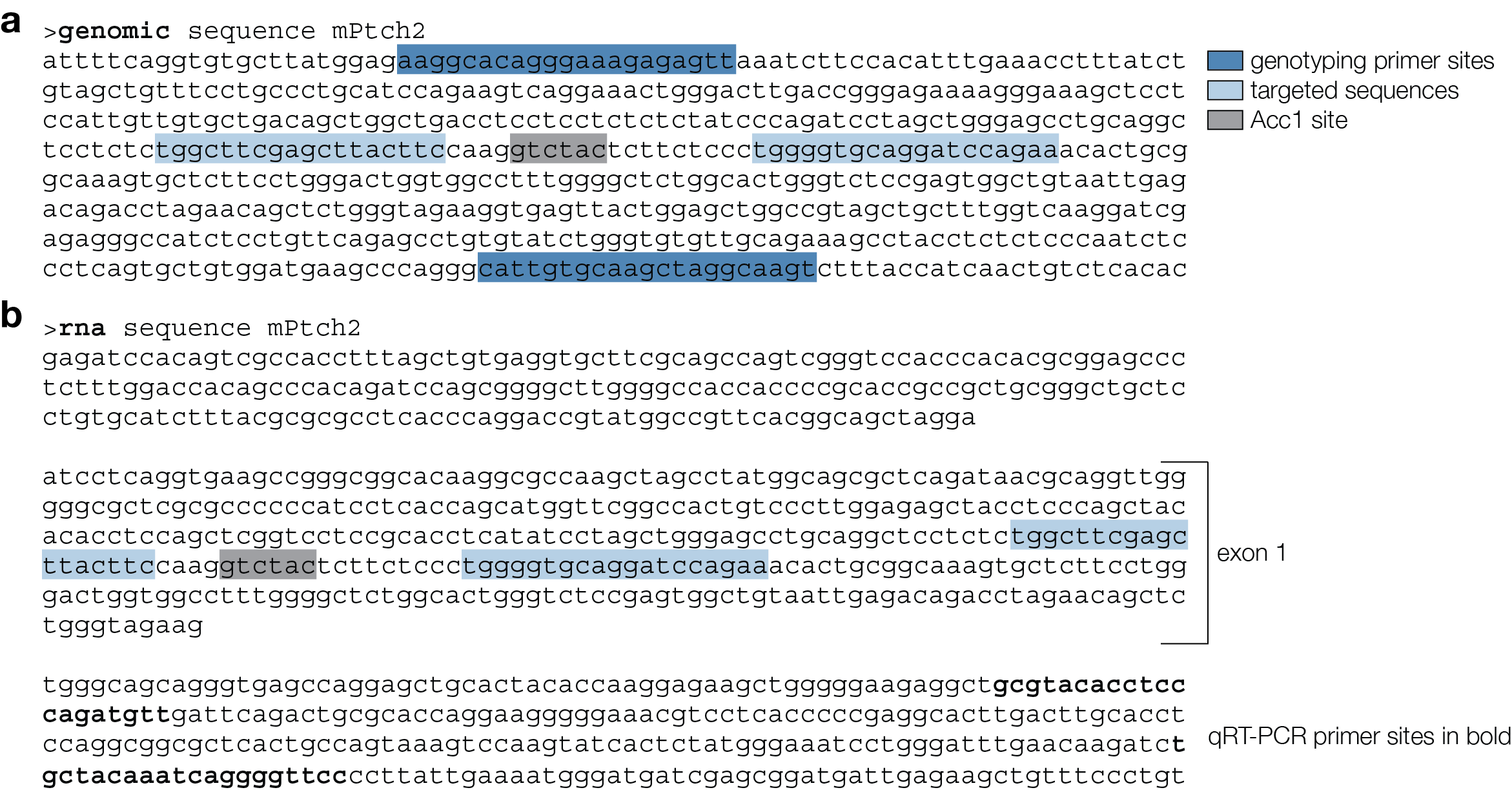
**

**Supplementary** **Fig. 4** Gene editing strategy and primer locations. (**a**) TALEN sequences were designed against the first exon of *Ptch2*. Target sequences for TALENs, sequencing primers, and restriction site for genotyping are color coded. (**b**) TALEN sequences in the RNA sequence around exon 1 are shown, and the qRT-PCR target sequences in bold.


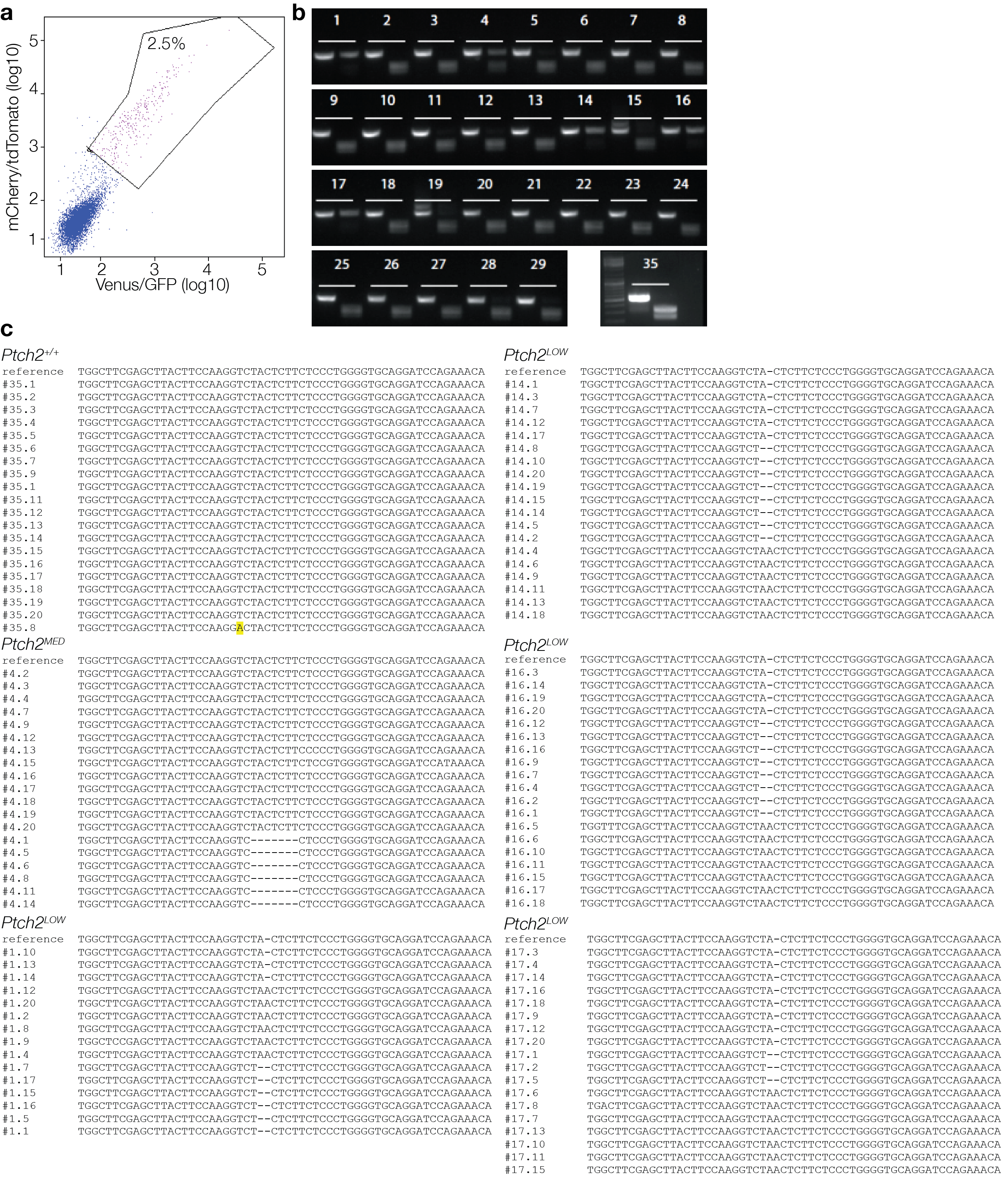


**Supplemental Fig. 5** Generation of ASZ001 TALEN cell lines. (**a**) ASZ001 cells were transfected with GFP/Tomato TALENs using PEI and after 72 h FACS-sorted using the gating strategy shown in the plot. Cells were plated in bulk and after 7 d, split into monocultures. See also Methods section. (**b**) Genomic DNA was isolated from TALEN-expressing ASZ001 monocultures and AccI restriction digests were performed to identify edited loci. Complete loss of enzyme activity is indicative of successful editing, see for instance line 1. (**c**) DNA from monoclonal lines with suspected editing events shown in panel b were TOPO cloned and 20 TOPO clones per cell line were Sanger sequenced. Shown are all successful sequencing reads. ‘Reference’ refers to current mouse genome build to which the Sanger sequences were aligned. In line 35, only wild type sequences were found. In line 4, approximately one third of the sequences did not align with the reference, hence the nomenclature *Ptch2^MED^*. For line 1, one third of sequences were found to be wild type and it was therefore called *Ptch2*^LOW^. For Line 14, 16 and 17 the same editing events were found as in line 1. In all these clones, the average fraction of wild type sequences was 0.29±0.06, insertion of an adenine was 0.37±0.02, deletion of an adenine was 0.35±0.08, suggestive of the presence of three *Ptch2* alleles in the ASZ001 cell line.


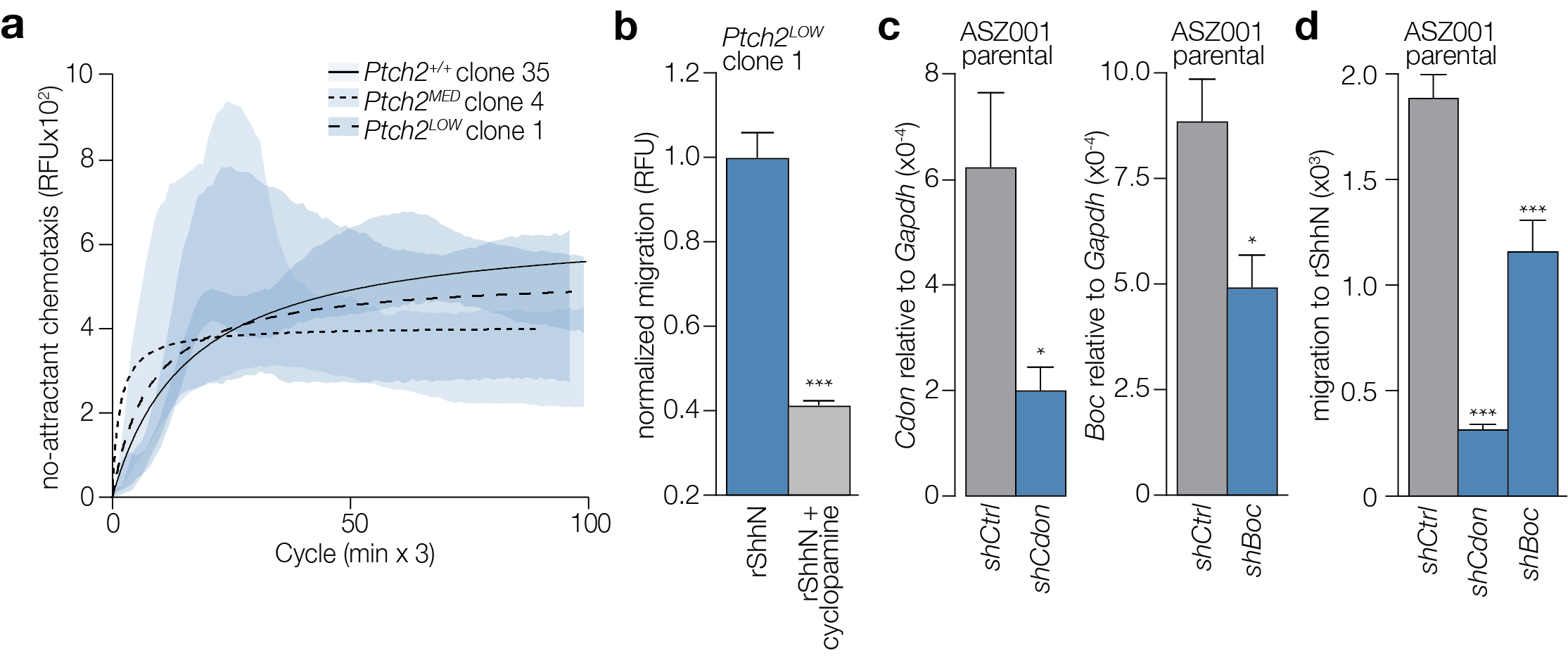


**Supplemental Fig. 6** Candidate receptors for Hedgehog ligand are required for Shh chemotaxis. (**a**) TALEN-edited ASZ001 cells were assayed in modified Boyden chamber migration assay as shown in Figure 3b. Migration to no-attractant control is shown. (**b**) *Ptch2^LOW^* cells were assayed for ShhN chemotaxis, with and without 5 µM cyclopamine in both upper and lower compartments of the Transwell setup. (**c**) ASZ001 parental cells were lentivirally transduced with TRC library clones and knockdown efficiency was assessed as for Supplementary Fig. 3. Shown are the most effective clones from 5 tested. (**d**) Chemotactic responsiveness to Shh was assessed as for Figure 2. *, p<0.05; *** p<0.001. These data were obtained in the parental, *Ptch2*-proficient, ASZ001 cell line.
